# Supplementary figures and images for: Host Porphobilinogen Deaminase Deficiency Confers Malaria Resistance in Plasmodium chabaudi but Not in Plasmodium berghei or Plasmodium falciparum During Intraerythrocytic Growth
Source: Front Cell Infect Microbiol. 2020 Sep 3;10:464. doi: 10.3389/fcimb.2020.00464 (PMC7495142; doi:10.3389/fcimb.2020.00464)

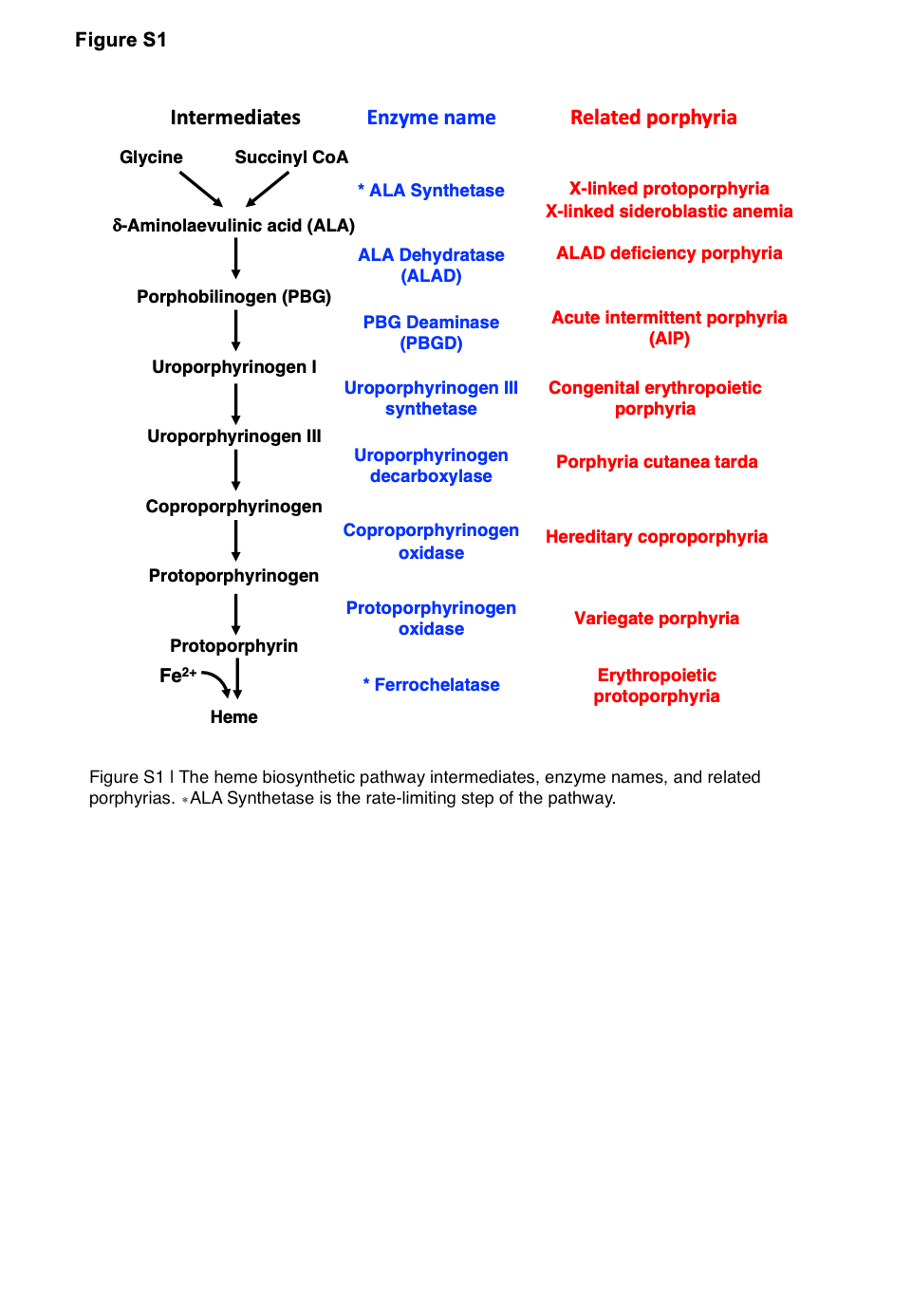

Supplement: Figure S1 — The heme biosynthetic pathway intermediates, enzyme names, and related porphyrias. *ALA Synthetase is the rate-limiting step of the pathway. [file Image_1.tiff]

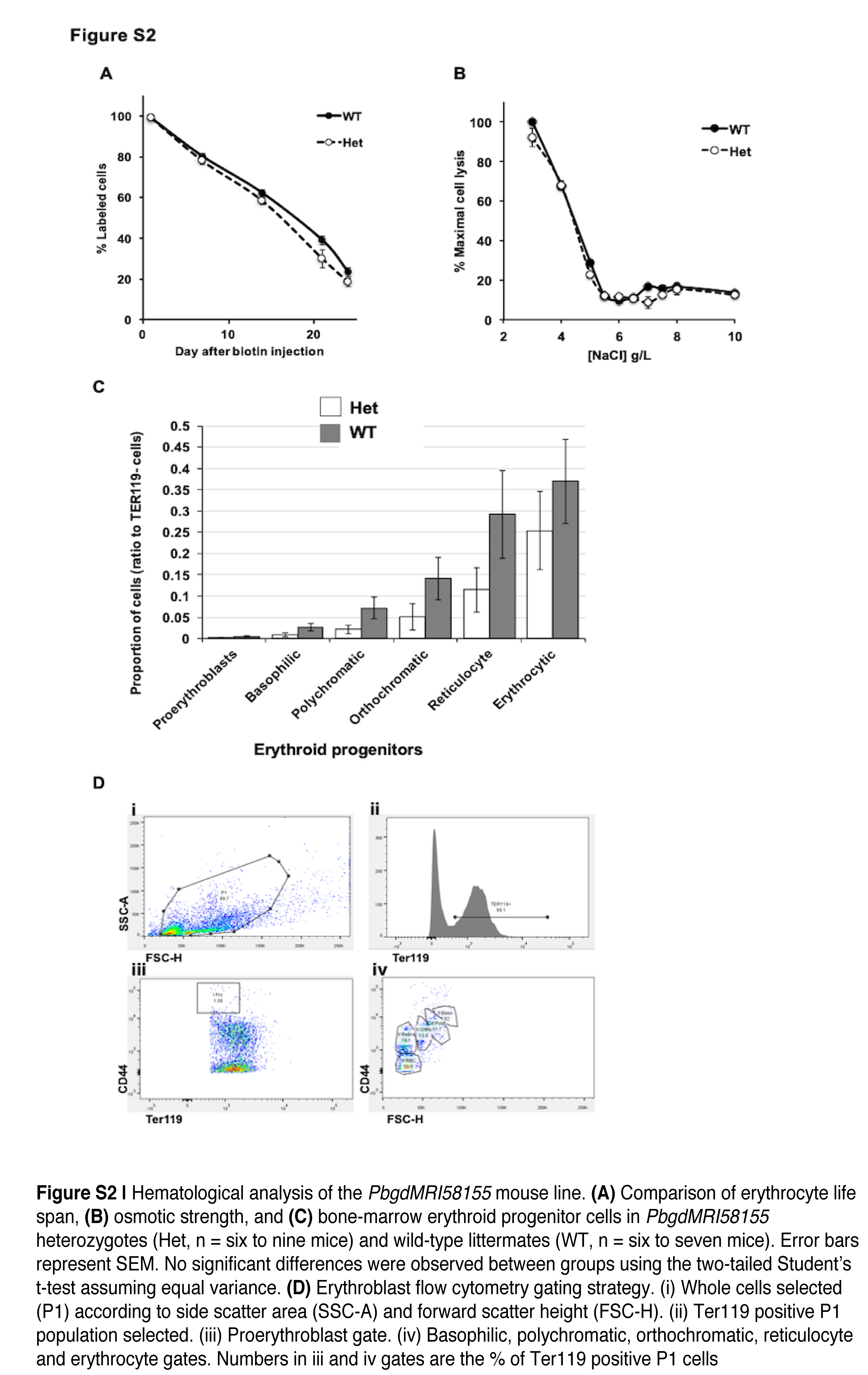

Supplement: Figure S2 — Hematological analysis of the PbgdMRI58155 mouse line. (A) Comparison of erythrocyte life span, (B) osmotic strength, and (C) bone-marrow erythroid progenitor cells in PbgdMRI58155 heterozygotes (Het, n = six to nine mice) and wild-type littermates (WT, n = six to seven mice). Error bars represent SEM. No significant differences were observed between groups using the two-tailed Student's t-test assuming equal variance. (D) Erythroblast flow cytometry gating strategy. (i) Whole cells selected (P1) according to side scatter area (SSC-A) and forward scatter height (FSC-H). (ii) Ter119 positive P1 population selected. (iii) Proerythroblast gate. (iv) Basophilic, polychromatic, orthochromatic, reticulocyte and erythrocyte gates. Numbers in iii and iv gates are the % of Ter119 positive P1 cells. [file Image_2.tif]

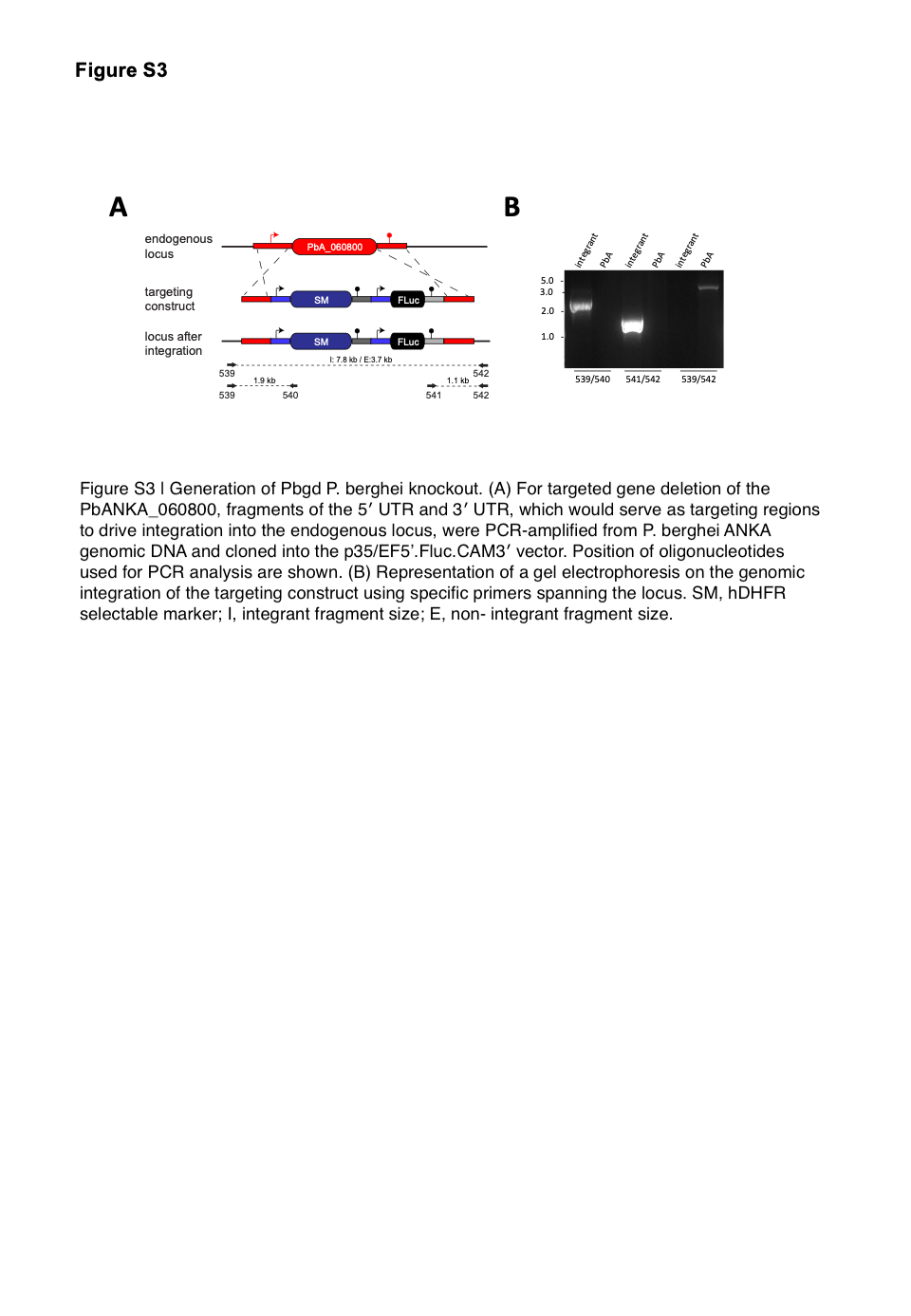

Supplement: Figure S3 — Generation of Pbgd P. berghei knockout. (A) For targeted gene deletion of the PbANKA_060800, fragments of the 5′ UTR and 3′ UTR, which would serve as targeting regions to drive integration into the endogenous locus, were PCR-amplified from P. berghei ANKA genomic DNA and cloned into the p35/EF5'.Fluc.CAM3′ vector. Position of oligonucleotides used for PCR analysis are shown. (B) Representation of a gel electrophoresis on the genomic integration of the targeting construct using specific primers spanning the locus. SM, hDHFR selectable marker; I, integrant fragment size; E, non- integrant fragment size. [file Image_3.tiff]

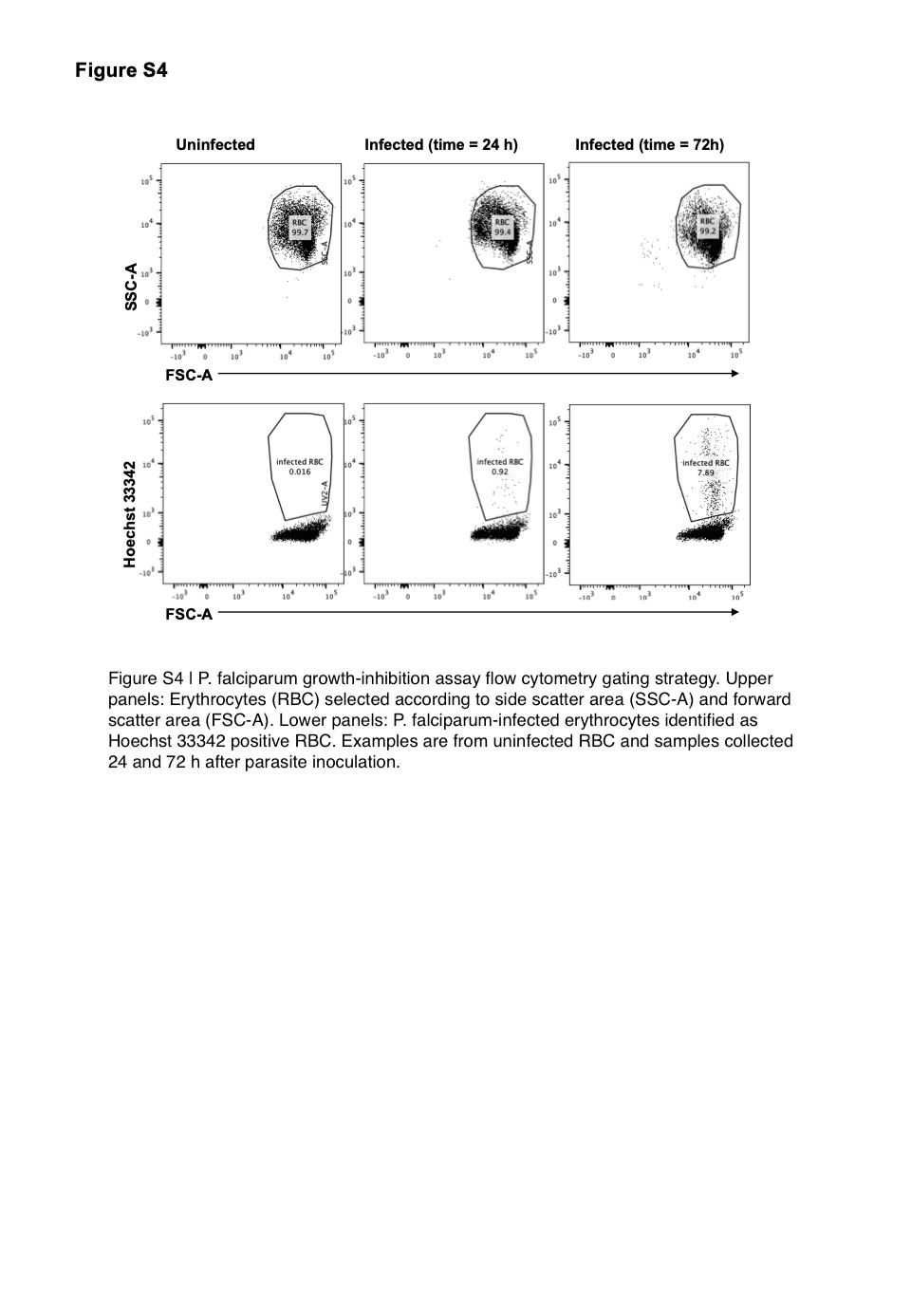

Supplement: Figure S4 — P. falciparum growth-inhibition assay flow cytometry gating strategy. Upper panels: Erythrocytes (RBC) selected according to side scatter area (SSC-A) and forward scatter area (FSC-A). Lower panels: P. falciparum-infected erythrocytes identified as Hoechst 33342 positive RBC. Examples are from uninfected RBC and samples collected 24 and 72 h after parasite inoculation. [file Image_4.tiff]
