# Supplementary material for: Host Porphobilinogen Deaminase Deficiency Confers Malaria Resistance in Plasmodium chabaudi but Not in Plasmodium berghei or Plasmodium falciparum During Intraerythrocytic Growth
Source: Front Cell Infect Microbiol. 2020 Sep 3;10:464. doi: 10.3389/fcimb.2020.00464 (PMC7495142; doi:10.3389/fcimb.2020.00464)
Supplement: Table S1 — Hematological parameters of wild-type and PbgdMRI58155 heterozygous mice. [file Table_1.docx]

**SUPPLEMENTAL TABLES**

**Table S1. Hematological parameters of wild-type and *Pbgd^MRI58155^*** **heterozygous mice.** Automated full blood analysis on SJL mice (n = 500), wild type (n = 24) and heterozygous (n = 63) mice. RBC = erythrocyte; WBC = white blood cell; MCV = mean corpuscular volume; HCT = haematocrit; % retics = proportion of reticulocytes; HGB = hemoglobin concentration; MCHC = mean corpuscular hemoglobin concentration. Values denote mean ± SEM.

** p < 0.01; *** p < 0.001.

|  | **RBC** | **WBC** | **MCV** | **HCT** | **Retics (%)** | **HGB (g/L)** | **MCHC (g/L)** |
| --- | --- | --- | --- | --- | --- | --- | --- |
| **Het** | 10.11 ± 0.17 | 11.09 ± 0.38 | 49.95 ± 0.22*** | 0.50 ± 0.008*** | 5.84 ± 0.26*** | 141.8 ± 2.5*** | 215.5 ± 7.4 |
| **WT** | 10.25 ± 0.05 | 11.44 ± 0.45 | 52.40 ± 0.23 | 0.54 ± 0.003 | 3.24 ± 0.21 | 153.4 ± 0.8 | 210 ± 6.5 |
| **SJL/J** | 9.79 ± 0.22 | 11.84 ± 0.50 | 52.61 ± 0.19 | 0.51 ± 0.011 | 3.44 ± 0.033 | 153.84 ± 0.23 | 203.53 ± 7.95 |

**Table S2. Spleen and liver indices, and non-heme iron content in wild-type and *Pbgd^MRI58155^*** **heterozygous mice (n = 3 per group).** Values denote means ± SD. * p < 0.05.

|  | **Spleen index** | **Liver index** | **Spleen iron (mg/g)** | **Liver iron (mg/g)** |
| --- | --- | --- | --- | --- |
| **Het** | 0.477 ± 0.017* | 5.8 ± 0.33 | 15.2 ± 1.2 | 3.59 ± 0.65 |
| **WT** | 0.408 ± 0.009 | 5.96 ± 0.16 | 11.4 ± 0.2 | 4.58 ± 0.62 |

**Table S3. Serum cytokine concentrations in wild-type (n = 5) and *Pbgd^MRI58155^*** **heterozygous (n = 8) mice. All cytokine concentrations are pg/mL plasma.** Values denote means ± SD. * p < 0.05.

|  | **IL-2** | **IL-4** | **IL-6** | **IFNγ** | **TNFα** | **IL-10** | **IL-17A** |
| --- | --- | --- | --- | --- | --- | --- | --- |
| **Het** | 119 ± 55 | 122 ± 56 | 84 ± 54 | 68 ± 42 | 140 ± 85 | 93 ± 42* | 110 ± 51* |
| **WT** | 192 ± 51 | 157 ± 56 | 156 ± 87 | 111 ± 53 | 186 ± 92 | 146 ± 34 | 187 ± 53 |

**Table S4. AIP patient blood biochemistry.**

| **AIP patient ID** | **ALA/Creatinine ratio** | **PBG/Creatinine ratio** | **Total urinary porphyrins (µg/L)** | **% Urine porphyrin types (URO, EPTA, ESA, PENTA, COPRO I, COPRO III)** | **PBGD activity (pmol/h/mg Hb)**** |
| --- | --- | --- | --- | --- | --- |
| **A** | 2.3 | 0.4 | 73 | 4, 1, <1, 10, 37, 48 | 61.1 |
| **B*** | 18.6 | 11.1 | 179 | 43, 3, 3, 22, 17, 12 | 74.8 |
| **C*** | 24.4 | 3.4 | 575 | 24, 2, 3, 23, 8, 41 | 56.9 |
| **D** | 1.5 | 0.3 | 88 | 8, 4, 1, 8, 58, 21 | 57.5 |

* Patients were symptomatic for AIP at the time of blood collection.

** Normal range: 72.8-179.6 pmol/hr/mg Hb
